# Supplementary material for: Intralayer and interlayer electron–phonon interactions in twisted graphene heterostructures
Source: Nat Commun. 2018 Mar 23;9:1221. doi: 10.1038/s41467-018-03479-3 (PMC5865138; doi:10.1038/s41467-018-03479-3)
Supplement: Supplementary file 1 — Supplementary Information(PDF 2759 kb) [file 41467_2018_3479_MOESM1_ESM.pdf]

Intralayer and interlayer electron-phonon interactions in twisted graphene heterostructures

G. S. N. Eliel[1,*], M. V. O. Moutinho[2,3,*], A. C. Gadelha[1], A. Righi[1], L. C. Campos[1], H. B. Ribeiro[4], Po-Wen Chiu[5], K. Watanabe[6], T. Taniguchi[6], P. Puech[7], M. Paillet[8], T. Michel[8], P. Venezuela[3], and M. A. Pimenta[1]

[1] *Departamento de Física, UFMG, Belo Horizonte, Minas Gerais, Brazil*
[2] *Núcleo Multidisciplinar de Pesquisas em Computação - NUMPEX-COMP, Campus Duque de Caxias, Universidade Federal do Rio de Janeiro, Duque de Caxias, RJ, Brazil.*
[3] *Instituto de Física, UFF, Niterói, Rio de Janeiro, Brazil*
[4] *Departamento de Engenharia Eletrica, Universidade Presbiteriana Mackenzie, São Paulo, SP Brazil*
[5] *National Tsing Hua University, Hsinchu, Taiwan*
[6] *Advanced Materials Laboratory, National Institute for Materials Science, 1-1 Namiki, Tsukuba 305-0044, Japan*
[7] *CEMES/CNRS, University of Toulouse, 31055 Toulouse, France*
[8] *Laboratoire Charles Coulomb, CNRS, Univ. Montpellier, Montpellier, France and*
[*] *G.S.N. Eliel and M.V.O. Moutinho contributed equally to this work.*

(Dated: February 13, 2018)

SUPPLEMENTARY NOTE 1: SAMPLE CHARACTERIZATION

The twisted bilayer graphene (TBG) samples were obtained via CVD method as detailed in Ref. 1, and transferred to different substrates: a 300 nm SiO_2/Si substrate for measurements in the visible range, 90 nm SiO_2/Si substrate for UV measurements, and fused silica for IR measurements. Each graphene layer presents a hexagonal shape with zigzag edges [1]. The twisting angle θ can be obtained from the analysis of the optical images, as shown in Supplementary Figures 1a,d. We can clearly see in the Raman maps represented in Supplementary Figs. 1b,c,e,f the different results for the 4° sample (Supplementary Figs. 1b,c) and the 13° samples (Supplementary Figs. 1e,f), excited with 1.92 eV (633 nm) and 2.33 (532 nm) laser lines, respectively. Notice in Supplementary Figs. 1c,f that the extra peaks appear only in the TBG regions.

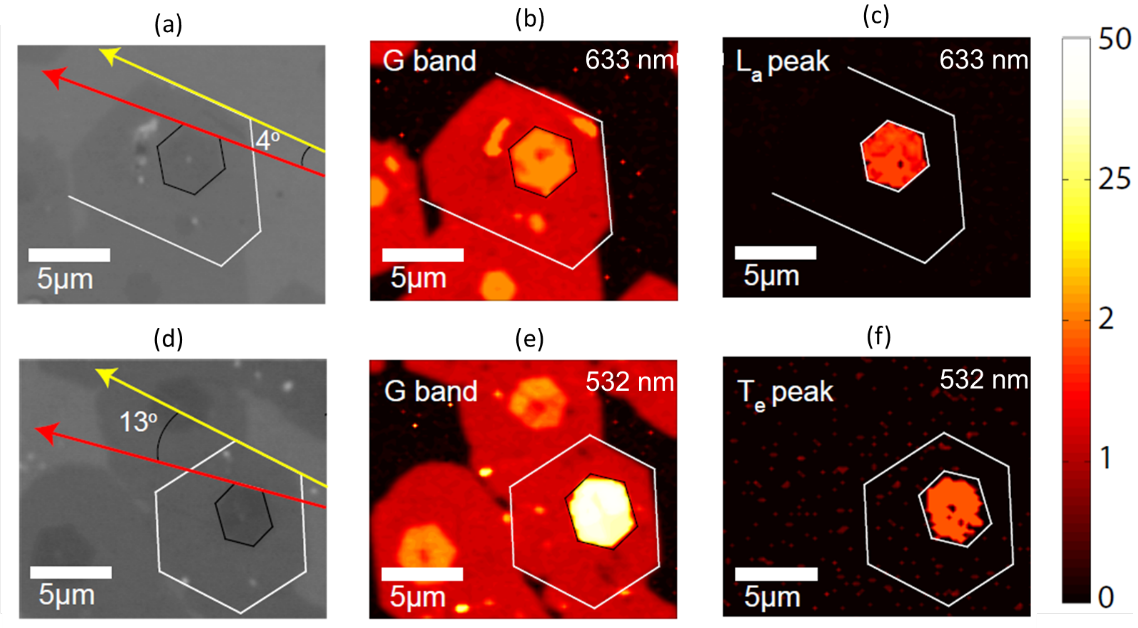

Supplementary Figure 1: (a,d) Optical images for two different samples used to obtain the twisting angle θ. (b,e) Raman maps of the G band for the samples shown in parts (a) and (d), respectively. (c) Raman map of the L_a peak and (f) Raman map of T_e peak.

The gr/BN samples were produced by mechanical exfoliation of graphene, which was then transferred to a h-BN substrate, providing the gr/h-BN heterostructure with a given twisting angle θ. Supplementary Figure 2a shows the AFM phase image of a gr/BN sample, where we can observe the h-BN and graphene flakes by contrast. Supplementary Figures 2b-d show Raman images of three different gr/BN samples. To obtain these images, we mapped the G band of graphene (in blue) and the h-BN peak at 1366 cm^{-1} (in red). In the region where h-BN

is covered with graphene, the image presents a purple colour. In these figures, the graphene and h-BN edges are highlighted by black and white lines, respectively. The twisting angles θ between graphene and h-BN edges were obtained from the analysis of the images and the values are shown in the Figures. The real angle can be θ or $\theta + 30$ due to the unknown orientation of the zigzag or armchair edges.

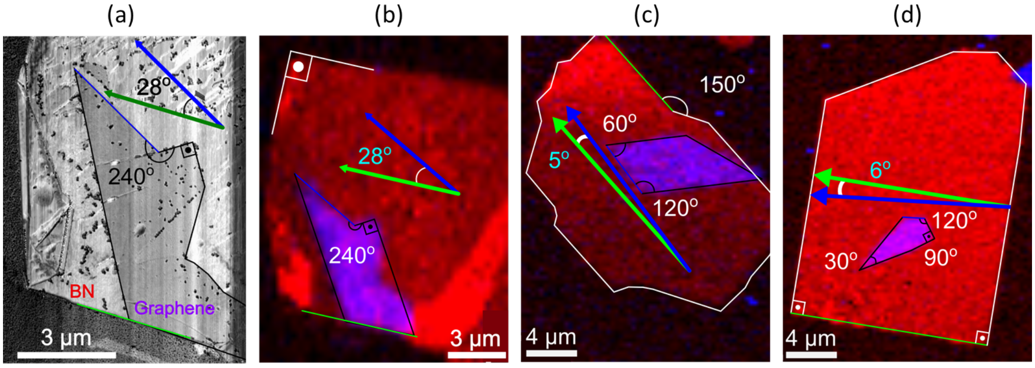

Supplementary Figure 2: (a) AFM image of a gr/BN sample (b-d) Raman maps of the G band (blue) and h-BN band (red) for three different gr/BN heterostructures, where the numbers in light blue represent the twisting angle between the crystallographic edges of graphene and h-BN.

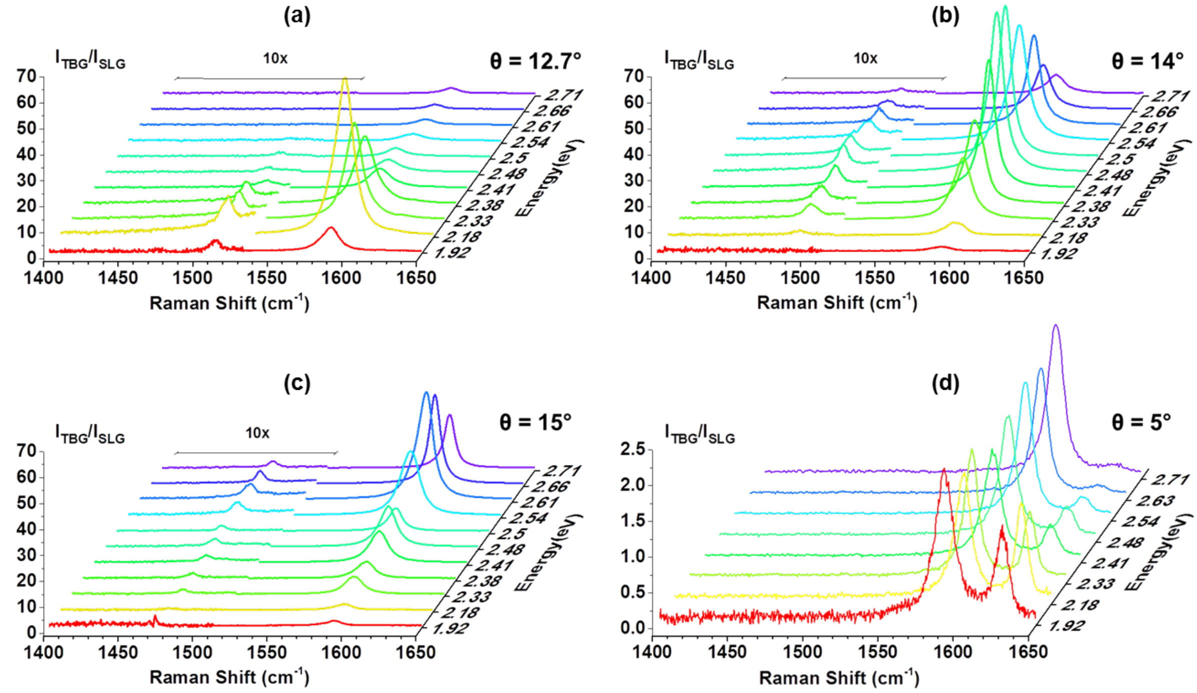

Supplementary Figure 3: (a-d) Raman spectra for four different TBG samples with twisting angles θ of 12.7°, 14°, 15° and 5°, respectively, using different laser lines in the visible range.

SUPPLEMENTARY NOTE 2: MULTIPLE EXCITATION RAMAN SPECTRA OF DIFFERENT SAMPLES USING VISIBLE LIGHT

For the multiple excitation measurements in the visible range, we used an Ar/Kr laser that provides many different excitation lines from 1.92 to 2.71 eV. Supplementary Figures 3a-c show the spectra for three samples in the interlayer resonance condition. In these cases, we can clearly observe the G band enhancement of almost 70 times and the appearance of the T_e peak in the spectral range of 1450 to 1530 cm^{-1}. Supplementary Figure 3d

shows the Raman spectrum of the sample with $\theta = 5°$ and the presence the L_a peak. The intensity of the G band is almost constant, whereas the L_a peak exhibits a resonance behaviour.

Supplementary Figure 4 shows the REP of the G band and the T_e peak in different TBG samples, normalised for the SLG intensity. The intensity of the T_e peak was multiplied by $\approx 100\times$ for comparison. The REP results for the L_a peak are presented in Supplementary Figure 5. The G band intensity is constant in this case.

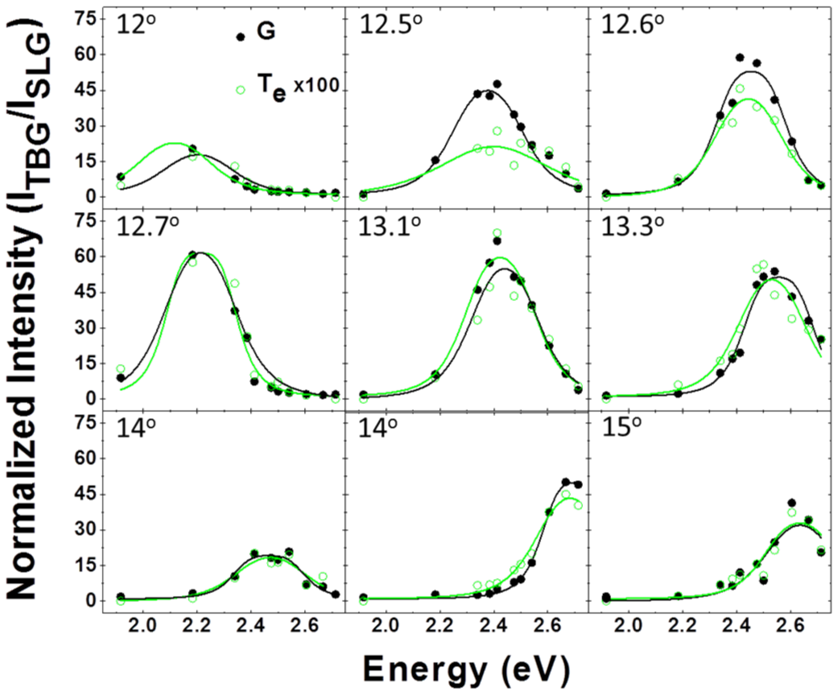

Supplementary Figure 4: Raman excitation profiles (REPs) of the G band (black circles) and T_e (green circles) peak in different samples. The values of θ for each sample are presented in the figures.

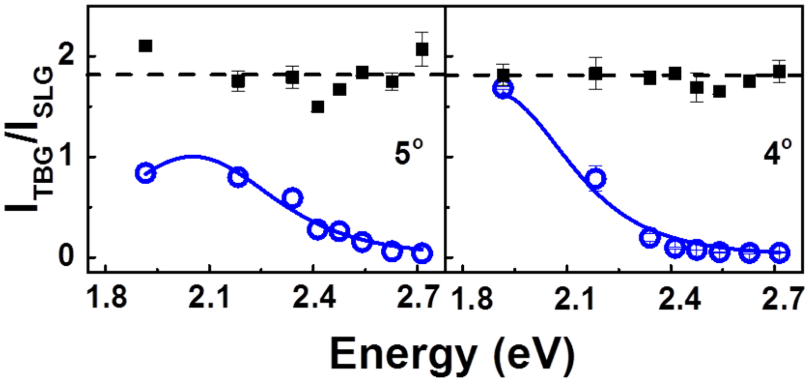

Supplementary Figure 5: Raman excitation profiles (REPs) of the the G band (black squares) and the L_a (blue circles) peak in two different samples. The dashed black line is the average relative intensity of the G band, around 1.8. The error bars represent the standard deviation.

SUPPLEMENTARY NOTE 3: ANALYSIS OF THE RAMAN EXCITATION PROFILES IN TWISTED BILAYER GRAPHENE

The full curves in Supplementary Figure 4 correspond to the best fit of the experimental data by the expression of the Raman-cross section based on the third-order perturbation process model, and given by:

$$I(E_L) = \left| \frac{K}{(E_{\rm L} - E_{\rm R} - i\Gamma)(E_{\rm L} - E_{\rm ph} - E_{\rm R} - i\Gamma)} \right|^2 \tag{1}$$

where $E_{\rm L}$, $E_{\rm R}$ and $E_{\rm ph}$ are the laser energy, the resonance energy and the phonon energy, respectively, and K is a constant related to the product of the transition matrix elements.

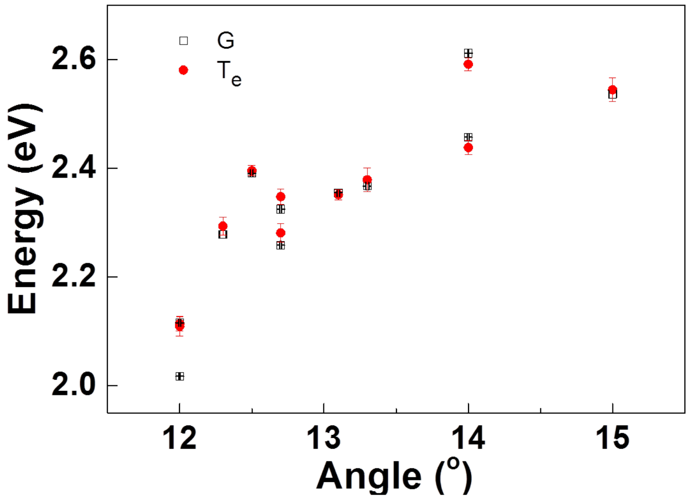

Supplementary Figure 6: Energy as a function of the twisted angle for G (black squares) and T_e (red circles) bands obtained via REP analysis. The error bars represent the standard deviation.

The values of $E_{\rm R}$ that fit the G and T_e bands shown in Supplementary Fig. 4 as a function of θ are presented in Supplementary Figure 6. Notice that the values of $E_{\rm R}$ for these two bands are practically the same, within the precision of the experiments, showing that they are enhanced by the same effect, which is the transition associated with the van Hove singularities. The analysis of the REPs of the G and T_e bands with Eq. 1 provides an accurate determination of the $E_{\rm vHs}$ values.

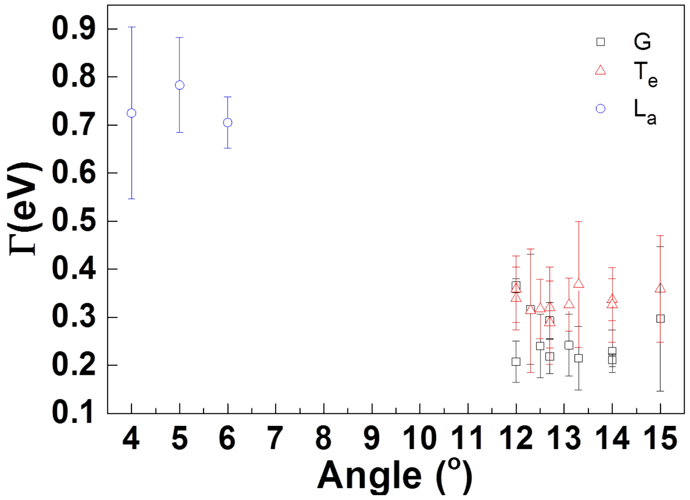

Supplementary Figure 7: REPs damping constant as a function of the twisting angle for G (black squares), T_e (red triangles) and L_a (blue circles) bands. The error bars represent the standard deviation.

Supplementary Figure 7 shows the values of the damping constant Γ that fit the data in Supplementary Fig. 4 as a function of the twisting angle. In the case we can observe a small difference for the G and T_e bands. In the case of G band (black squares), the average value of the damping constant Γ is around 250 meV, but for

T_e (red triangles) this value increases, being around 330 meV. Considering that both processes are first-order Raman process in the reduced Brillouin zone, we can conclude that this difference arises from the different electron-phonon interaction, that decreases the photo-excited electron lifetime. For the intralayer case, the width Γ of resonance is around 0.7 eV and also represented by the blue circles in Supplementary Figure 7.

SUPPLEMENTARY NOTE 4: RAMAN SPECTRA OF DIFFERENT SAMPLES USING INFRARED AND VISIBLE LIGHT

For the Raman measurements performed in the infrared and red ranges, three laser lines were used: 680, 730 and 830 nm (1.82, 1.70 and 1.49 eV, respectively). In this case, we used a fused silica substrate to avoid interference effects. Supplementary Figure 8 shows all results for samples that present the (a) interlayer and (b) intralayer el-ph processes. Due to the few number of laser lines, no resonance profile can be obtained. Therefore, the data presented in Figure 3 of the main article correspond to the laser line energy of the spectrum where the Raman peaks exhibit the maximum enhancement.

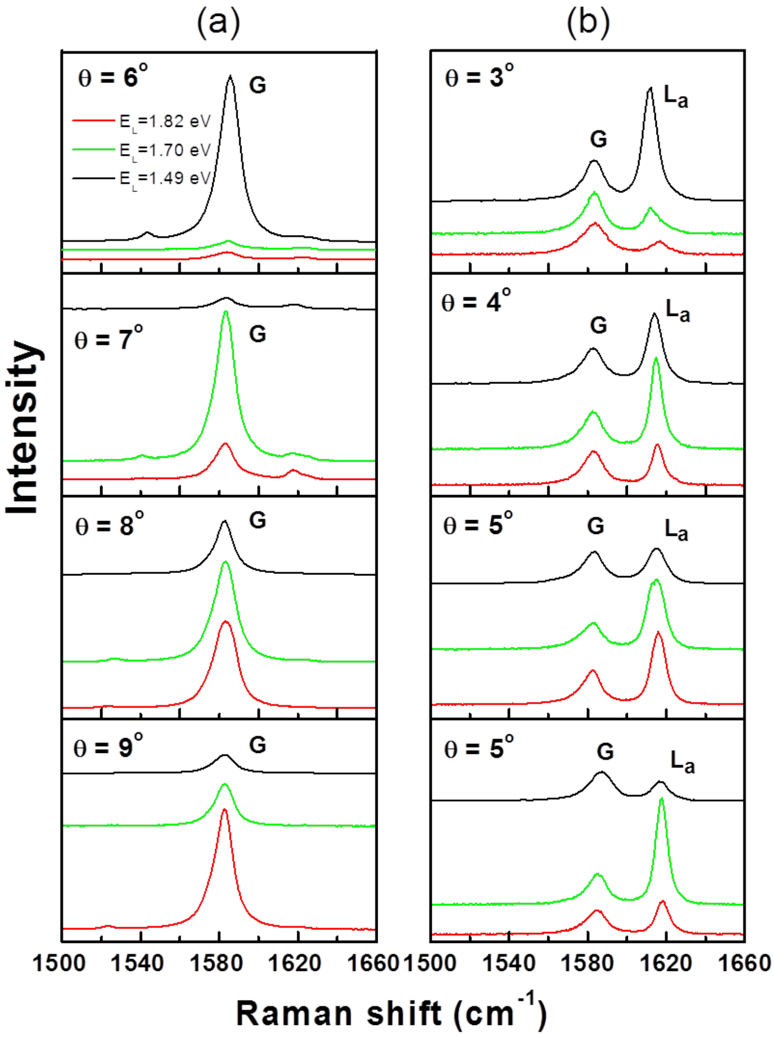

Supplementary Figure 8: Raman spectra for samples with low twisting angle θ excited using IR and near red excitation. (a) Interlayer case, (b) intralayer case.

SUPPLEMENTARY NOTE 5: RAMAN INTENSITIES OF INTRALAYER AND INTERLAYER MODES

Supplementary Figure 9 shows the relative intensities of T_a, T_e L_a and L_e as a function of the twisting angle θ. Notice that they increase with decreasing values of θ.

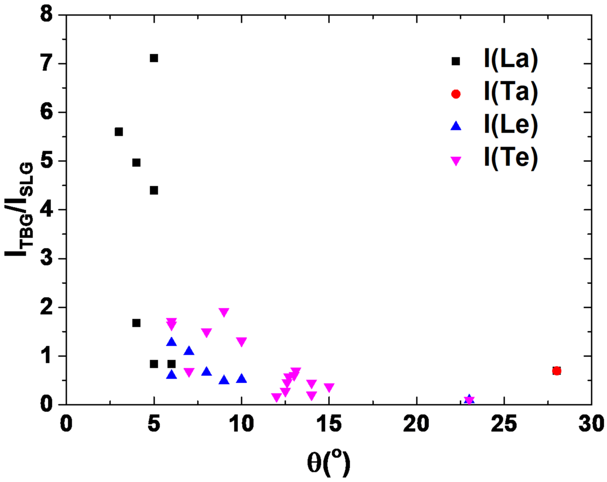

Supplementary Figure 9: Relative intensities of the T_a, T_e L_a and L_e peaks as a function of the twisting angle.
.

[1] C.-C. Lu, Y.-C. Lin, Z. Liu, C.-H. Yeh, K. Suenaga, and P.-W. Chiu, ACS Nano **7**, 2587 (2013), pMID: 23448165, http://dx.doi.org/10.1021/nn3059828.
